# Credit constraints and rural farmers’ welfare in an agrarian economy

**DOI:** 10.1016/j.heliyon.2020.e05252

**Published:** 2020-10-14

**Authors:** Amanullah, Ghulam Rasool Lakhan, Siraj Ahmed Channa, Habibullah Magsi, Mansoor Ahmed Koondher, Jing Wang, Naseer Ahmed Channa

**Affiliations:** a Department of Economics, Abdul Haq Campus, Federal Urdu University of Arts, Science & Technology, M.A Jinnah Road, Karachi, 75300, Pakistan; b Department of Plant Breeding and Genetics, Sindh Agriculture University, Tandojam, 70060, Sindh, Pakistan; c Department of Agricultural Economics, Sindh Agriculture University, Tandojam, 70060, Sindh, Pakistan; d College of Economics and Management, Northwest A&F University, Yangling, Shaanxi, 712100, China; e Bank Alfalah Ltd, Jamshed Road, Branch, Karachi, Sindh, Pakistan

**Keywords:** Credit constraints, Farmer's welfare, Agrarian economy, Sindh, Pakistan, Agricultural economics, Economics, Management, Human resource management

## Abstract

This study aims to determine the impact of credit constraints on wheat farmers' welfare. For this study, data on 575 wheat farmers were collected through a simple random sampling technique. The treatment-effect model was used to find the effect of credit constraints on the farmers' welfare. In addition, to control for the problem of endogeneity, ordinary least squares and logistic regressions were employed. Farmers' welfare was measured by as consumption. The results show that constrained farmers cultivate 2.8–4.1% more area of land than unconstrained farmers but that the spending and income per capita of credit-constrained farmers are 18.9 to 13.8% lower, respectivley, than those of unconstrained farmers. Moreover, the results indicate that the welfare and income of credit-constrained farmers are influenced by age, the interest rate, area of land, and family size. An increase in the area of land enhances wellbeing and returns for the constrained farmer, which is in contrast to the unconstrained farmer. The results show that credit constraints have a negative impact on farmers' welfare and income. Better welfare may only be achieved if credit is supplied to credit-constrained farmers. Furthermore, this study has potentially significant implications. First, the negative impact of the interest rate suggests that the State Bank of Pakistan should modify agricultural credit policies, particularly to design a flexible interest rate for farmers. Second, the central bank should amend the agricultural credit limits based on the current financial needs of the agricultural market when the rate of inflation is high and the impact of agricultural crises is long and drawn-out. Third, the government should launch an agricultural Islamic bank in the study area. This Islamic bank would support religious farmers who are constrained due to objections to interest, and it would relax the farmers’ credit constraints as well as help them to increase welfare and income.

## 1 Introduction

Agriculture is a significant source for the livelihood of population all over the world which plays a pivotal role in poverty reduction and hunger minimization. Farmers face a lot of hardship such as minimum productivity from labor, low profits, and credit constraints that have adverse effects on farm output. Farm productivity mainly relies on credit factors for securing agricultural land. Agricultural credits widely improve farmers' income and welfare. To promote farmers' welfare, most of the agrarian countries including Pakistan targeted the potential gains of farmers through credit programs (Amanullah et al., 2019a). The Agrarian economy is originated in 1960 to provide social relations with dynamic production and reproduction property. It also offers a change in the historical and contemporary process for the formation and development of agriculture. Further, it provides drastic economic development of modern history through different agricultural paths and farm production at different times and places (Bernstein, 2015). Notwithstanding, a mismatch in the capital market provides a negative impact on farm growth (Popov, 2014). In modern technology, agricultural credit is considered as an essential factor for farm productivity. The agrarian economy is used to secure agricultural credit, in percentage term it is about 85% of a total credits (Kumar et al., 2017). Usually, the small scale farmers use agricultural credit for their survival and large scale farmers use that to improve their income streams (Das et al., 2009) Van-Vugt et al. (2018) confirmed that wheat farmer's income not only depends on the farming business but also on the access of agricultural credit sources. Similarly, Ngeno (2018), and Solano and Rooks (2018) considered both access to credit and socioeconomic attributes for the enhancement of farm household well-being.

In developing economies, farmers are usually hindered by credit constraints and insufficient income because they are unable to provide collateral for bank credits (Conning and Udry, 2007). In Pakistan, the rural credit market incorporates both formal and informal reforms for a substantial role in the rural economy (Saqib et al., 2018). Formal credit uses district panel data and credit with increased rural productivity and income (Kumar et al., 2017). Government credits were common before the 1990s for farmers with strategic default (Adjognon et al., 2017). For the effective functioning of farm productivity, internal or external financing constraints play critical roles. The combination of external and internal financing factors affects significantly on farm productivity, and due to insufficient cash flow many farms demand external finances (Li et al., 2018). The slow growth process is evident in least developed countries due to pervasive credit constraints and poor financial system (Skott and Gómez-Ramírez, 2018). When the farmers are unable to get credit at lower interest rates they ultimately get credit at high interest rate. It is worth nothing that the repayment of credit is a big task if the credit is inherited. This study is an attempt to investigate the credit constraints face by the farmers and what possible sources they can use to get hassle free credits.

## 2 Literature review and hypotheses development

### 2.1 Credit constraints and farmers welfare

The credit constraints mainly influence farmers output, investment, income, and welfare. Amanullah et al. (2019a) showed the adverse effects of credit constraints on farmer's income and investment. The study concluded that the income and investment of farmers could be increased by 7.2% and 5.1% respectively with the minimization of credit constraints. Also, the access to credit portrays a crucial function in the expansion of income and investment. The results explain that the credit-constrained farmers had low income and investment than unconstrained farmers. Similarly, Ciaian et al. (2012) demonstrate that the credit constraints not only reduce agricultural production but also slow-down economic growth in remote areas. Tang et al. (2010) found that rural household savings and spending influenced by credit access. As a result, access to credit, empowers farmer's potential to carry out his budgetary necessities that facilitate to smoothly invest in inputs, implements, and productive investment. Moreover, Jia et al. (2010) found that the credit-constrained restricted farmers in wealth creation actions.

The scarcity of agricultural economic goods and insufficient access to credit might affect farmer's well-being, which consists of farm production, sustenance, and food security (Ali and Awade, 2019; Awotide et al., 2015; Asiedu et al., 2013). Farmers could procure farm implements, but they are urged by credit constraints to confine their spending and production preferences (Dong et al., 2012). Coleman (1999) and Li et al. (2011) defined credit constraint as the policy failure and inadequate access to formal credit loans were considered as credit constrained, which mostly hinders farmers from enhancing their living standard, well-being, and increasing farm production. Baiyegunhi et al. (2010) indicate that credit constraints have a negative effect on farmers' welfare, and the farmers who are relaxed from credit constraints have relatively higher monthly spending as compare to credit-constrained farmers. Tran et al. (2016) discovered that credit constraints limit the consumption expenses of credit-constrained farmers while unconstrained farmer's consumption expenses were unlimited. Kumar et al. (2013) imply that the food and health expenditure, farm implements and academic achievement of Chinese and Indian farmers hindered by credit constraints. Li and Zhi (2010), and Dong et al. (2010) explained that credit constraints decrease income by 13.2% in China, and elimination of credit constraints can enhance about 23.2% of income.

A very few studies have been conducted to estimate the effects of credit constraints on income in Pakistan either at regional levels or in various cropping regions (Amanullah et al., 2019a; Elahi et al., 2018; Chandio et al., 2017; Mehmood et al., 2017). To the best of our knowledge, there is no research carried out to estimate the impact of credit constraints on farmer's income/welfare especially in Sindh region of Pakistan. Therefore, this study is unique in nature to determine the effects of credit constraints on wheat farmer's income/welfare in consequences of utility purposes (spending and income) in the Sindh province of Pakistan. By the help of an in-depth analysis of previous literature we use spending as the indicator for welfare which is also used by Wossen et al. (2017), Tran et al. (2016); Muayila and Tollens (2012), and Baiyegunhi et al. (2010) A recent study by Ali and Awade (2019) utilized production and net revenue as an indicator to measure farmer's welfare.

Keeping in view the hurdles in accessibility of agricultural credits from the formal sources, this study aims to investigate the relationship between credit constraints and farmers’ income/welfare by using the cross-sectional data with advanced econometric techniques like the treatment effect model to estimate the effects of credit constraints, then, the ordinary least square and logistic regression techniques to address the endogeneity issues. The following are the hypotheses of this study:

H_0_ Credit constraints have no effects on wheat farmers' welfare and income.

H_1_ Credit constraints have significant effects on wheat farmers' welfare and income.

### 2.2 Wheat production in Pakistan

Pakistan occupies the 8^th^ position in the world and third position in Asia based on wheat cultivation, production volume, and yield per hectare (PARC, 2018). Wheat is the most important cereal crop and the main resource of high-calorie intake for the people in different shapes. It is considered as the essential diet in the countryside. A vast majority of the growers produce wheat on 80% area which is approximately nine million hectares, and consist of nearly 40% agricultural land of Pakistan (Amanullah et al., 2019b). The contribution of wheat is 1.7% in the growth of the agriculture sector and 9.1% in the GDP growth. The wheat was grown about 9,734 thousand hectares in 2017–18 indicating a decline of 2.6% as compares to 8,072 thousand hectares last year. The wheat production shows a small decrease of 4.4%–25.5 million tons in the year 2017–18 compared to 26.6 million tons in the year 2016–17 (GOP, 2018). Alongside with other important factors in decreased production such as climate changes, expensive inputs, and heavy rainfall, the key reason was a lengthy and cumbersome procedure of provision of agriculture credit from formal credit sources (Koondher et al., 2018).

## 3 Materials and methods

### 3.1 Study area and sampling method

**Figure 1:**
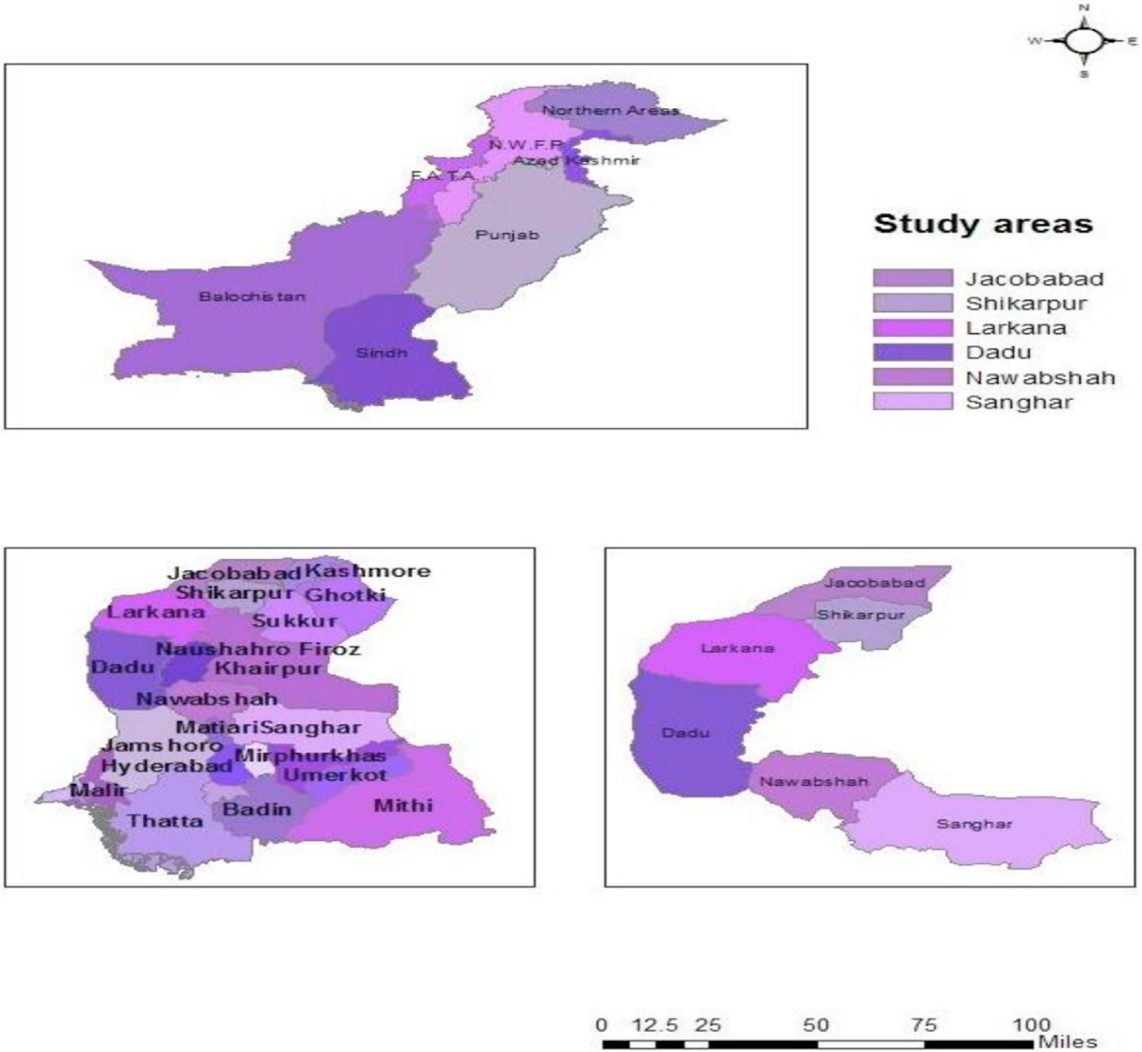
Study area in Sindh Pakistan.

This study was carried out in the Sindh province of Pakistan in 2017. The survey data of 575 respondents were collected from the six major wheat-growing districts namely, Dadu, Larkana, Jacobabad, Shikarpur, Nawabshah, and Sanghar (Figure 1). From each district, four administrative subdivisions (Taluka's) were selected. Two union councils were chosen from each Taluka. In Pakistan, a union council (UC) explains a municipal administration in the country, and each union council is comprised of the different villages (Abid et al., 2016). Two villages were randomly selected from each union council based on ease of data collection; six wheat-growing respondents were interviewed by the help of the Agriculture Extension and Agriculture Department, of Sindh Government, Pakistan.

#### 3.1.1 Constraints from formal institutions

The land was used as a significant factor for obtaining credit in rural areas of the Sindh province of Pakistan. The factors considered for evaluation are farm size, total production, mode of expenses, and productivity from particular land in the selected area. This research uses data collected from 575 sample populations all have experience of farming activity and facing credit constraints in obtaining credit for wheat cultivation. The analysis was carried out by considering demographic variables, farmland, asset factors, productivity, and other factors involved in wheat production. Finally, the treatment effect analysis was carried out to evaluate credit constraints influencing factors in selected regions of Pakistan. Data were collected under how the distance, loan amount, interest rate, loan procedure, and religious activity affect the formal credit constraints. On the other hand, factors involved in managing informal credit were also evaluated for selected farm area. In this research, the entire analysis is performed with considering land as a collateral factor for rural area development and credit constraints.

#### 3.1.2 Collateral used by the respondents for the agricultural loan

The credit to a particular individual farmer is denied or is limited due to inadequate collateral. Usually, a number of methods are used for collateral such as agricultural land, commercial property, landlord guarantee, the guarantee offered by the neighbor or relatives, and any other source. Most of the farmers bound their lands for collateral in the bank with a negligible share. A farmer mainly relies on collateral for increasing tenant access and rural household towards institutional credit. The strict requirement of collateral by formal credit institutions increases the credit constraints. Adequate collateral was a crucial issue in obtaining loans from formal credit organizations in the rural areas of Sindh province (see Table 1).

**Table 1:** Description and summary statistics of variables.

Variables	Measurement unit	Mean	(Std. Dev)
Age	Age of farmer in years	44.51	10.80
Landownership	I If farmers have landownership	0.94	0.22
Interest rate	Interest paid on loan (in percentage)	8.77	8.96
Area of land (log)	Land cultivated in hectares	4.35	5.08
Family size	Number of family members	10.45	4.27
Access to extension	1 if accessed extension services	0.58	0.49
Labour	Income per labor measured in logarithm	8.83	0.02
Dadu	1 If farmer settled in Dadu	0.16	0.37
Shikarpur	1 if farmer settled in Shikarpur	0.16	0.37
Jacobabad	1 if farmer settled in Jacobabad	0.17	0.37
Nawabshah	1 if farmer settled in Nawabshah	0.17	0.37
Sanghar	1 if farmer settled in Sanghar	0.16	0.36
Income	Income earned from wheat in Rs. 1000/season	226543	682163
Spending	Total food and consumption expense in Rs. 1000/month in wheat season	384927	229620
Collateral	1 if credit institution required collateral	0.37	0.48
Credit constraints	1 if farmer is credit constrained	0.49	0.50
Number of observations		575	

#### 3.1.3 Formal institutions for credit

**Figure 2:**
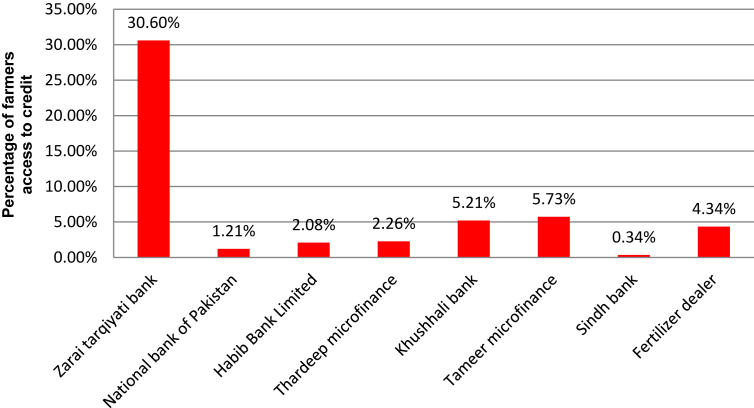
Farmers' access to credit (data source: Field survey 2017).

Data collected from the sample are evaluated for borrowing channel performance. The sample data expressed that the following organizations offered credit to farmers in the rural area of Pakistan (Figure 2). The 30.60% of farmers obtained credit from the Zarai Taraqiati Bank. Tameer Microfinance occupies the second position for offering credit with 5.73% while Khushhali bank occupies the third position with 5.21%. However, the majority of farmers (48%) do not have any idea about borrowing channels. The analysis concluded that the majority of the population is not aware of the borrowing channels.

#### 3.1.4 Identification of credit constraints

In the light of previous studies conducted by Zhao et al. (2014), and Boucher et al. (2009), we utilized the following consequences to identify the credit-constrained status of farmers:

i. A farmer's credit application refused by the formal credit organization is measured as total quantity rationing.
ii. A farmer obtained an insufficient amount (less than 50%) is considered as partly quantity rationing.
iii. A farmer rejected the credit institution's offer due to high-interest rate, and other loan charges are considered as price or transaction cost rationing.
iv. A farmer did not submit a credit application because he feared that it would lose land or collateral is considered as risk rationing. Similarly, a farmer is said to be unconstrained he preeminently obtained credit.

The analysis of collected data explained that in almost all selected regions of sample credit constraints are evident which is a main hurdle in the growth of an agrarian economy. In case of credit constraints categorization, total quantity rationing involves 2.27%, partly quantity rationing 1.0%, and transaction cost or price rationing comprises 16.17% of the total population. Finally, risk-rated credit constraints accounted for 30.2% of the total population count. In this study, out of 575 respondents, 259 households (45.0% of farm households) applied for formal credit, while 316 households (54.9% of farm households) have not applied for formal credit. There are 286 households 49.7% of farm households are credit constrained (Table 2).

**Table 2:** Association between credit constraints and application status.

Credit application status	Farm individual who applied for formal credit		Farm individual who did not applied for formal credit			Total %
	Number	Percentage %	Number		Percentage %	
Number of farmers	259	45.0	316		54.9	575 (100.0)
Constrained	286	49.7				

### 3.2 Econometric specification of model

This study depends on the hypothesis that credit constraints influence the farmer's spending and income. We begin by presenting a structure of individual spending and credit constraints then we use it in the welfare and income of the farmers. Similar to the methods used by Jappelli (1990), Diagne and Zeller (2001), and Sawada et al. (2006), we constructed response method of endogenous credit constraints by representing the existence of credit constraints cc. We assume that a farm individual devours a certain quantity of commodities, C, in a given period time. Let C∗ represent the best spending in the lack of credit constraints. C∗ = C (the real spending) if the credit constraint is not restricting; C∗ > C if the credit constraint is limiting. The space between the optimum levels of spending and real spending finds the presence and absence of credit constraints. We suppose that the wheat farmers spending difference is defined as $Spending_{wht}^{*}\;=C-C^{*}$. Following the studies of Jappelli (1990), Gilligan et al. (2005), and Sawada et al. (2006), two components find whether a farmer will face credit constraints or not? The first component is the demand for credit, which is the variation between individual income and necessary spending. The second component connects to the supply of credit by financial organizations. The optimum spending C∗ and the maximum accessible credit to the individual both can be described as a linear function of discernible like the human capital and physical capital. The simple form of spending gap equation can be expressed as:

(1) $$Spending_{Wht}^{*}=\omega z+\mu$$

$$cc\;=1\;if\;s^{*}<0\;otherwise\;0\;if\;s^{*}\;\geq 0$$

where ω explains farm individual and farm features that identify demand and supply of credit to the farmers; μis a random error with zero means. A farm individual is said to be restricting credit constraint if $Spending_{wht}^{*}<0\;and\;hence\;cc=1$. The credit constraint is not restricting if $Spending_{wht}^{*}\geq 0\;$and therefore cc = 0. The empirical impact of credit constraint on the welfare and income can be constituted of two dependent variable models. The first model is a credit constraint Eq. (1). The second model associates to the welfare and income that the endogenous credit constraint condition of a farm individual is entailed as an independent variable as shown in the next equation:

(2) $$Y_{spending\;wht}=\delta_{spendigwht}+{\text{Χ}}_{Z}^{\iota }\gamma_{Z}+\upsilon$$

(3) $$Y_{income\;wht}=\eta_{incomewht}+{\text{W}}_{Z}^{\iota }\beta_{Z}+\varepsilon$$

where *Y*_*spendingwht*_ and *Y*_*incomewht*_ are the wheat household's spending and income ${\text{Χ}}_{Z}^{\iota }\;and\;{\text{W}}_{Z}^{\iota }$ is a matrix of household-specific socioeconomic and demographic characteristics that affect the farmer's spending and income. The last term υ,ε are the error. The disturbance terms υ,ε have zero mean, bivariate normal distribution with unit variance and ρ=corr(υ,ε). The covariate matrix is written as follows:

$$\left[\begin{array}{cc} \sigma & \rho \\ \rho & 1 \end{array}\right]$$

Green (2000) proposed that if μ and ε are connected, then the estimation of equation (Eqs. (2) and (3)) is inconsistent for α and β. We observed that the credit needs of all the farmers are not similar, such as credit-constrained and unconstrained farmers possess different landholdings (small, medium and large landholding). For instance, some farmer's do not need credit, whereas others need credit, but needy could not obtain credit due to constraints. Therefore, the evaluation of the impact of credit constraints and its determinants is less likely to suffer from selection bias. When estimating the effect of credit constraints, issue of endogeneity will arise from discreet factors, which affects the farmer's participation in credit access and their credit constraints status (Amanullah et al., 2019a; Dong et al., 2012). For example, all farmers are not homogenous; that is why some have additional funds or did not need credit. In this condition, the effect of credit constraints possibly will be biased for that reason. To estimate the impact of credit constraints on welfare and income, we employed the treatment effects model. It is examined the effect of an endogenous dual treatment *cc* on an extended fully observed variable *y*, tentative on the independent variables *x* and *w*. The prime importance is in the regression function (Eqs. (2) and (3)). In the preferred treatment model, *cc* is the endogenous dummy variable signifying whether the treatment is accredited or not. The binary result treatment *cc* is created as the result of an ignored latent variable *cc∗*. It is presumed that *cc∗* is a linear function of the exogenous covariate *w* and a random factor *u*. Further, to conclude the issue of endogeneity and selection bias, we run the additional regression by using ordinary least square and logistic regression to get the exact outcome from selection bias.

## 4 Results and discussion

### 4.1 Credit constraint in access to credit

**Figure 3:**
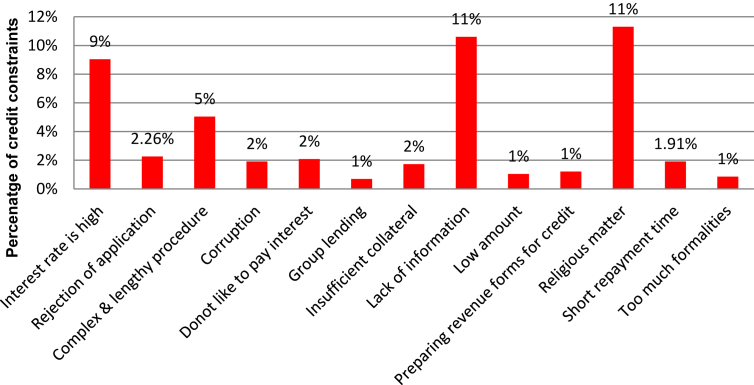
Credit constraints in access to credit (Data source: field survey, 2017).

Several factors are involved in access to credit, which shows a barrier for prospective applicants in obtaining credit (Figure 3). The remaining sample population count 11% opted that religious matter is stopping criteria for credit constraints in banks. This outcome is in line with Saqib et al. (2016), and Amanullah et al. (2019a) as their studies accepted that in the context of Muslim society in Pakistan, farmers do not like to take loans on interest as it is forbidden in the religion (Islam). Lack of information and interest rate occupies second and third position towards credit constraints factors. This result is similar to Assogba et al. (2017), and Ololade and Olagunju (2013) as their studies confirmed that farmers could not avail credit facility due to high-interest rates. Other factors, such as complex and lengthy procedure, rejection of application, corruption, do not like to pay interest, insufficient collateral are considered as lesser influencing factors of credit constraints. The finding of these factors is line with Amanullah et al. (2019a); Elahi et al. (2018), and Hussain and Thapa (2012) as their studies accepted that farmers are credit constrained due to these factors. It is concluded that religious thinking, lack of information, and higher interest rate are significant factors for credit constraints (Figure 3).

### 4.2 Estimates of treatment effect model

The coefficient of age is positive and significant at 5% level of significance. It indicates that if the age of farmers increases the possibility of credit constraints increases. Possibly the older farmers have more experience in farming, and they know that if more amount is invested in farming, the output will be high. So, the risk of credit constraints decreases. Likewise, formal financial institutions have confidence in older farmers due to their excellent creditworthiness and credit experiences. The result of our study is reliable with previous studies conducted by Freeman et al. (1998); Barslund and Tarp (2008) and Porgo et al. (2018). The variable for land ownership is significant which, indicates that when landownership decreases the probability of credit constraints increases. However, the variable land ownership is not significant (Table 4) which shows no effect on the income of constrained wheat farmers. The coefficient of interest rate is negative and statistically significant at 1% in welfare and income equation. The negative factor explains that if interest rate increases the likelihood of getting loans decreases. It implies that farm households were anxious about the high-interest rate. It suggests that the negative impact of interest rate dampen farmers in obtaining loans from formal credit institutions. According to Saqib et al. (2016) farmers who do not like to pay interest did not take loans from the formal credit institute due to Riba-Prohibition in Islam. This outcome is constant with several studies like (Amanullah et al., 2019a; Assogba et al., 2017; Ololade and Olagunju, 2013; Saifullahi and Haruna, 2012; Mpuga, 2004) as their results showed that farm households were vulnerable to take advantage from credit programs owing to costly interest rates. The coefficient for the variable area of land is positive and statistically significant at the level of 1% in both equations. It implies that an increase in the area of land increases the probability of credit constrained. Further, it explains that large farm growers have more possibilities of being credit constrained because large farm size needs more amount for purchasing inputs and needs to hire more labor to complete the work on time. However, the findings of our study are in line with (Lin et al., 2019; Kuwornu, 2013; Jia et al., 2010) as their studies exposed that area of land significantly influences the credit constraint's.

The magnitude of variable family size is positive and statistically significant at the level of 1% in the welfare equation (See Table 3) while in the income equation, it is insignificant. It indicates that an increase in a farmer's family size increases the probability of credit constraints as the credit constraints influence family expenditure positively. This study found that 51% of the farmers have a medium-sized family, and 38% have a large family. The medium family-size farmers have the highest share of credit-constrained farmers (49.66%) compared to the small and large farmers. Similarly, the studies carried out in Burkina Faso, Eastern Region of Ghana, Nigeria, and Pakistan explains that an increase in family size will increase the likelihood of farmers being credit constraints. Our results are similar to Saqib et al. (2018), and Oyedele et al. (2009). The four regional variables for Shikarpur, Jacobabad, Nawabshah, and Sanghar, found negative and significant in the welfare equation which indicates that the credit constraints have a negative relationship with farmers' welfare in these regions. Whereas in the income equation, two regional variables Nawabshah and Sanghar, were significant indicating the highest impact of credit constraints on income of wheat farmers in these regions.

**Table 3:** Maximum Likelihood Estimates for welfare.

	Treatment Effect		OLS Regression	Logistic Regression
	Credit constrained	Full sample	Full sample	Full sample
	Coefficient (Std. Err.)	Coefficient (Std. Err.)	Coefficient (Std. Err.)	Coefficient (Std. Err.)
Age	0.001 (0.0006)∗	0.0103 (0.004)∗	0.0002 (0.0005)	0.035 (.041)
Landownership	-0.041 (0.033)	-0.738 (0.26)∗∗∗	-0.013 (0.025)	1.184 (1.381)
Interest rate	-0.331 (0.005)∗∗∗	-0.134 (0.047)∗∗∗	-0.289 (0.005)∗∗∗	-3.629 (.672)∗∗∗
Area of land (Log)	0.028 (0.010)∗∗∗	0.347 (0.082)∗∗∗	-8.37e (0.008)	-0.308 (.500)
Family size	0.042 (0.016)∗∗∗	0.577 (0.119)∗∗∗	0.004 (0.012)	0.946 (1.042)
Access to extension	0.0117 (0.014)	-0.025 (0.113)	0.004 (0.011)	-0.209 (.837)
Dadu	-0.146 (0.025)∗∗∗	-0.953 (0.193)∗∗∗	-0.050 (0.019)∗∗	-1.467 (1.109)
Shikarpur	-0.131 (0.025)∗∗∗	-0.840 (0.192)∗∗∗	-0.048 (0.019)∗∗	-2.313 (3.473)
Jacobabad	-0.106 (0.024)∗∗∗	-0.284 (0.187)	-0.088 (0.018)∗∗∗	-4.813 (1.630)∗∗∗
Nawabshah	-0.114 (0.024)∗∗∗	-0.771 (0.185)∗∗∗	-0.054 (0.019)∗∗∗	-3.738 (2.074)∗
Sanghar	-0.119 (0.025)∗∗∗	-0.887 (0.192)∗∗∗	-0.044 (0.019)∗∗∗	-1.504 (1.359)
Spending	-0.189 (0.013)∗∗∗	-	0.024 (0.120)∗∗	2.139 (0.989)∗∗
Collateral	-	0.656 (0.122)∗∗∗	-0.178 (0.015)∗∗∗	-5.082 (1.393)∗∗∗
Constant	0.776 (0.055)∗∗∗	-0.401 (0.286)	1.015 (0.029)∗∗∗	2.139 (0.989)∗∗
/Athrho∗		1.220 (0.064)∗∗∗		
/lnsigma	-1.783 (0.035)∗∗∗			
Rho		0.839 (0.019)∗∗∗		
Sigma		0.168 (0.005)∗∗∗		
Chi2 = 105.85	LR test of independence equations:Prob > Chi2 = 0.000			

Standard Errors are reported in parenthesis as. ∗∗∗, ∗∗and ∗denotes 1, 5 and 10 statistically significant levels.

∗lnsigma and Athrho are transformations of sigma and rho that are used in' the estimation process. Source: Field survey 2017.

**Table 4:** Maximum Likelihood Estimates for income.

	Treatment Effect		OLS Regression	Logistic Regression
	Credit constrained	Full sample	Full sample	Full sample
	Coefficient (Std. Err.)	Coefficient (Std. Err.)	Coefficient (Std. Err.)	Coefficient (Std. Err.)
Age	-.00001 (0.0006)	-0.006 (0.005)	0.0005 (0.0005)	0.047 (0.039)
Landownership	-0.016 (0.030)	-0.314 (0.281)	-0.015 (0.025)	0.952 (1.376)
Interest rate	-0.329 (0.004)∗∗∗	-0.162 (0.060)∗∗∗	-0.289 (0.005)∗∗∗	-3.393 (0.586)∗∗∗
Area of land (Log)	0.041 (0.011)∗∗∗	1.376 (0.139)∗∗∗	0.007 (0.009)	-0.501 (0.594)
Family size	-0.003 (0.014)	0.006 (0.153)	0.001 (0.011)	0.932 (0.998)
Access to extension	0.006 (0.013)	-0.071 (0.144)	0.005 (0.011)	-0.014 (0.839)
Dadu	-0.075 (0.022) ∗∗∗	0.178 (0.211)	-0.061 (0.019)∗∗∗	-2.185 (1.185)∗
Shikarpur	-0.066 (0.023) ∗∗∗	0.121 (0.260)∗∗	-0.059 (0.019)∗∗∗	-3.099 (3.153)
Jacobabad	-0.110 (0.022)∗∗∗	-0.446 (0.210)∗∗∗	-0.086 (0.018)∗∗∗	-4.525 (1.565)∗∗∗
Nawabshah	-0.028 (0.022)	0.715 (0.233)∗∗∗	-0.071 (0.019)∗∗∗	-4.437 (1.946)∗∗
Sanghar	-0.015 (0.023)	0.991 (0.264)∗∗∗	-0.063 (0.019)∗∗∗	-2.430 (1.230)∗∗
Income	-0.138 (0.019)∗∗∗	-	0.036 (0.015)∗∗	1.807 (1.039)∗
Collateral	-	0.711 (0.171)∗∗∗	-0.178 (0.015)∗∗∗	-4.667 (1.278)∗∗∗
Constant	1.089 (0.035)∗∗∗	-0.668 (0.333)∗∗	1.004 (0.029)∗∗∗	6.967 (2.256)∗∗∗
/Athrho∗		0.911 (0.092)∗∗∗		
/lnsigma	-1.886 (0.033)∗∗∗			
Rho		0.721 (0.044)∗∗∗		
Sigma		0.151 (0.005)∗∗∗		
Chi^2^ = 29.08	LR test of independence equations:Prob > Chi2 = 0.000			

Standard Errors are reported in parenthesis as. ∗∗∗, ∗∗and ∗denotes 1, 5 and 10 statistically significant levels.

∗lnsigma and Athrho are transformations of sigma and rho that are used in' the estimation process. Source: Field survey 2017.

### 4.3 Impact of credit constraints on welfare and income

The estimated coefficient for age is positive and significant in the welfare equation. It indicates that older farmers have more farming experience than younger farmers and older farmers manage their farming and family spending expense effectively to smooth their living standards. In the income equation, the variable age is insignificant showing no effect of age on the income of farmers. This result is consistent with Li et al. (2013) that explained that age did not influence the farmer's income. The variable interest rate is highly significant at 1% level of significance in both welfare and income equations. It shows the negative and significant effect on the welfare and income of wheat farmers. It suggests that the highest impact of the interest rate dampen farmers in obtaining loans from formal credit institutions. This outcome is similar to Amanullah et al. (2019a) and Mehmood et al. (2017) as their results confirmed that formal credit sources charge high interest on loans and that is why farmers are constrained in obtaining loans from formal credit sources.

The variable area of land is positive and significant in both welfare and income equations. It impacts the farmers’ livelihood positively and significantly. Furthermore, it implies that as the area of land increases the capacity of income, when income increases the welfare of farming households will automatically increase. The outcome of this variable is similar to previous studies like (Wossen et al., 2017; Abdulai, 2016). The coefficient of family size is positive and significant in the welfare equation. Possibly, it explains two reasons for its significance. First, the farmers who have a large family size; they do not need to hire more labor because family members work as family labor, which decreases the cost of labor and increases the income. Second, large family farmers have more possibilities of earning income from other off-farm activities. Therefore, family size has a positive effect on the welfare of farmers. This result is in line with Lin et al. (2019) as their results explained that one unit increase in family size would raise 11.82% consumption of farm household. The all-geographical variables Dadu, Shikarpur, Jacobabad, Nawabshah, and Sanghar, are highly significant at 1% level of significance in the welfare equation. They are showing the negative impact of credit constraints on the welfare of wheat farmers in the selected areas. Whereas in the income equation, only three regional variables Dadu, Shikarpur, and Jacobabad are significant, further, showing the highest impact of credit constraints on the income of wheat farmers in these areas. It implies that credit constraints have a massive effect on the welfare and income of the wheat farmers. The findings of the treatment effect determine the strong influence of credit constraints on the welfare and income of wheat farmers in the study area. The variables interest rate; area of land and family size have the greater effects on the welfare and income of farmers. This findings indicates a dire need to eradicate the credit constraints from study areas. To relax farmers from the credit constraints, the government should revise the agricultural credit policies regarding interest rate and amount of credit.

Besides, the significance of the correlation coefficient (Rho) is statistically significant in both models which imply that the sample may suffer from selection bias and treatment estimation might give biased outcomes. It indicates that credit constraint is endogenous and therefore, we cannot reject the null hypothesis for no endogeneity of credit constraint condition of a farmer. It shows that credit constraints have a negative and significant impact on farmers' consumption. Further, it explains that unconstrained farmer spending was above average and better than constrained farmers. This outcome implies that credit constraint decreases the consumption of growers. It is similar to our predictions that owing to credit constraints wheat growers are restricted for smoothing their spending. This finding is consistent with Lin et al. (2019) and Tran et al. (2016) as their results show severe impacts of credit constraints on farmer's spending.

### 4.4 Robustness check

The result is emergent as it can be utilized as a robustness check for the strength of the treatment effect model. The constant significant impacts of credit constraints on the interest rate and area of land in both economic consequences (See Tables 3 and 4) highlight the important function that adequate finance in agriculture business might play a role in increasing the welfare and income of wheat farmers. Further, to check the additional robustness of the model. We follow Tran et al. (2016) to utilize income per labor as the substitute for farmers’ welfare and income. The result is reported in Tables A1 and A2. The likelihood ratio of welfare with chi-square x^2^(2) = 117.98 and for income with chi-square x^2^(2) = 24.33 both are significant at 1% implies that the endogenous relationship between wheat farmers credit constraint condition and their income per labour. The correlation coefficients Rho and Rho1 in Table A1 both are significant and positive at a 1% level only for wheat farmers spending that is unconstrained, signifying that farmers who are relaxed from credit constraints contain higher than normal spending in contrast to a random farmer in the model. While the correlation of a coefficient (/Athrho) is positive and significant at the 1% level in Table A2, which explains that unconstrained credit, farmers have higher income per capita than credit-constrained farmers. The significance of collateral signifies that the role of collateral in obtaining loans from formal credit sources for unconstrained farmers. Furthermore, it suggests that those farmer lack collateral which mostly places farmers in the umbrella of credit constraints and restricted their income creating measures.

## 5 Conclusions

Our analysis indicates that the welfare of credit-constrained farmers influenced by age, interest rate, area of land, and family size, while the income of credit-constrained farmers influenced by interest rate and area of land. The positive and significant impact of the age of the farmer on welfare emphasizes that agricultural credit must be intended to old growers who are credit constrained to increase their welfare. This study suggests that formal credit organizations should launch some agricultural and credit utilization workshops for old growers to aware of modern farming techniques; and new agricultural financing products for the development of farmer's welfare and income in the study region.

The results of this study are interesting that constrained farmers cultivate more area of land 2.8–4.1% than the unconstrained, but spending and income per capita of a credit-constrained farmer is lower than the unconstrained farmer 18.9 to 13.8% respectively (see Tables 3 and 4). However, other studies such as Wossen et al. (2017), and Abdulai (2016) found that area of land increases the production, income, and consumption capacities of farm households. Moreover, adequate credit can reduce the welfare lacks of constrained farmers. In the previous literature, Lin et al. (2019); Phan (2012) found that adequate and full amount of credit increases the welfare and income of constrained farmers 1.35 and 1.32% respectively. In the light of above outcomes, our study presents essential suggestions to formal credit organizations that credit-constraints in the studied region are vulnerable to farm households. To enhance the income and welfare of farmer's credit constraints must be removed from the rural credit market.

Moreover, the outcome of this research implies that credit administration can play a significant role in country's output, and investment can increase production and household welfare. The treatment effect findings entail that there could be a massive influence in supplying additional credit to the credit-constrained groups for eliminating the constraints through access to adequate credit. Additionally, if formal credit access could be upgraded, it may facilitate farmers to buy the optimal level of inputs and smooth their spending. The majority of farmers do not have access to formal credit as; they are living under the dilemma of being credit constrained. In this situation, constrained free and quick access to credit intended to credit-constrained farmers will help to increase their income and welfare.

## 6 Implications, limitations and future research paths

The significant results of this article indicate that consumption and income may be very important in enhancing the level of rural farmers' welfare in an agrarian economy. The findings from this study present potential significant implications. First, the agriculture sector is the backbone of a country's economic development, and it provides food and employment in the country. The majority of people are engaged in agricultural businesses. Therefore, it is recommended that agricultural credit policies should be especially tailored to facilitate flexible interest rates for farmers. In Pakistan financial institutions charged high interest rates, which mostly increased credit constraints (Elahi et al., 2018). Thus, the negative impact of the interest rate is due to high interest rates. Formal credit organizations should decrease the interest rate to the benefit of farmers and reduce the cost of credit to encourage farmers to access agricultural credit. The low-interest rates are easy to pay and are more convenient when utilized for farming as a longer period of time is required to produce a crop. It is important to explain that farmers might be unable to fully take advantage of the agricultural credit provided by formal credit organizations. Second, the central bank should amend the size of agricultural credits according to the current financial needs of the agricultural commodities when the rate of inflation is high and according the duration and velocity of the agricultural impacts of crises. Finally, the findings explained that the harmful impacts of credit constraints were larger on the welfare and income of the agriculture sector. Interest-free credit schemes or Islamic financing mechanisms might be instruments that are effective in reducing the negative effect of the interest rate in agricultural financing. Islamic financing products provide perfect techniques that are pertinent to agriculture and rural financing to develop the economy. They not only provide a feasible outcome but also constitutes have a significant effect on the welfare of farmers and people. Furthermore, Shafiai and Moi (2017) confirmed the significant and positive impact of Islamic financing products on the welfare of poor farmers in Malaysia. Certainly, welfare and income in the agrarian economy depend on agricultural production, while farmers in developing nations expend most of their earnings on consumption. Therefore, the government should launch an interest free banking (Agricultural Islamic Bank) in the study area. Also, Islamic banking institutions would encourage religious beliefs among farmers who are constrained due to interest to protect them from credit constraints and help them increase their welfare and income.

The study has a limitation of the unattainable facts and figures at the provincial and country-level and inaccessibility of panel data. A comprehensive study could be carried out utilizing utilizable data with supplementary variables of panel data sets in the future. Another limitation of this study is that the data were collected from only six major wheat-producing districts of Sindh. In-depth data could be collected from all wheat-growing districts at the country level. Furthermore, to find the influence of credit constraints on the agriculture sector at the aggregate level. It could be interesting if a future study carries out on the economic welfare of cash and horticulture crop growers in a whole part of the country.

## Declarations

### Author contribution statement

Amanullah: Conceived and designed the experiments; Analyzed and interpreted the data; Wrote the paper.

G.R. Lakhan, M.A. Koondher, S.A. Channa and N.A. Channa: Analyzed and interpreted the data; Contributed reagents, materials, analysis tools or data.

J. Wang: Conceived and designed the experiments; Wrote the paper.

H. Magsi: Analyzed and interpreted the data.

### Funding statement

This research did not receive any specific grant from funding agencies in the public, commercial, or not-for-profit sectors.

### Competing interest statement

The authors declare no conflict of interest.

### Additional information

No additional information is available for this paper.

## Figures and Tables

**Table A1:** Maximum Likelihood Estimates for welfare.

	Treatment Effect		OLS Regression	Logistic Regression
	Credit constrained	Full sample	Full sample	Full sample
	Coefficient (Std. Err.)	Coefficient (Std. Err.)	Coefficient (Std. Err.)	Coefficient (Std. Err.)
Age	0.0018 (0.0006)∗∗∗	0.0123 (0.0049)∗∗	0.0002 (0.0005)	-0.5351 (0.0467)
Landownership	0.0314 (0.0336)	0.5692 (0.2533)∗∗	0.0144 (0.0255)	-1.3621 (1.4196)
Interest rate	-0.3321 (0.0050)∗∗∗	-0.1486 (0.0472)∗∗∗	-0.2890 (0.0053)∗∗∗	-3.4971 (0.8306)∗∗∗
Area of land (Log)	0.0287 (0.0117)∗∗	0.3151 (0.0866)∗∗∗	0.0005 (0.0089)	-0.1989 (0.5822)
Access to extension	0.0134 (0.0151)	-0.0008 (0.1114)	0.0041 (0.0114)	-0.5751 (.8920)
Labour	0.0109 (0.0132)	0.1906 (0.0974)∗	-0.0022 (0.0100)	-1.1214 (0.7794)
Dadu	-0.1552 (0.0256)∗∗∗	-1.0533 (0.1900)∗∗∗	-0.0498 (0.0198)∗∗	-1.3260 (1.1194)
Shikarpur	-0.1349 (0.0258)∗∗∗	-0.8611 (0.1902)∗∗∗	-0.0482 (0.0198)∗∗	-2.8009 (4.0630)
Jacobabad	-0.1118 (0.0248)∗∗∗	-0.3674 (0.1848)∗∗	-0.0880 (0.0188)∗∗∗	-5.0850 (1.6804)∗∗∗
Nawabshah	-0.1114 (0.0250)∗∗∗	-0.7207 (0.1825)∗∗∗	-0.0552 (0.0190)∗∗∗	-3.9837 (2.0257)∗∗
Sanghar	-0.1199 (0.0259)∗∗∗	-0.8499 (0.1908)∗∗∗	-0.0448 (0.0197)∗∗	-1.6476 (1.4763)
Spending	-0.1922 (0.0134)∗∗∗	---	0.0236 (0.0117)∗∗	2.6816 (1.1304)∗∗
Collateral	---	0.6891 (0.1201)∗∗∗	-0.1789 (0.0159)∗∗∗	-5.1889 (1.3667)∗∗∗
Constant	0.9391 (0.1217)∗∗∗	-2.5221 (0.8991)∗∗∗	1.0197 (0.0922)∗∗∗	18.6442 (7.6401)∗∗
/Athrho∗		1.2459 (0.0636)∗∗∗		
/lnsigma	-1.7735 (0.0358)∗∗∗			
Rho		0.8471 (0.0179)∗∗∗		
Sigma		0.1697 (0.0060)∗∗∗		
Chi2 = 117.98	LR test of independence equations:Prob > Chi2 = 0.000			

Standard Errors are reported in parenthesis as. ∗∗∗, ∗∗and ∗denotes 1, 5 and 10 statistically significant levels.

∗lnsigma and Athrho are transformations of sigma and rho that are used in' the estimation process. Source: Field survey 2017.

**Table A2:** Maximum Likelihood Estimates for income.

	Treatment Effect		OLS Regression	Logistic Regression
	Credit constrained	Full sample	Full sample	Full sample
	Coefficient (Std. Err.)	Coefficient (Std. Err.)	Coefficient (Std. Err.)	Coefficient (Std. Err.)
Age	-0.000013 (0.0005)	-0.0059 (0.0059)	0.00057 (0.00050)	0.0559 (0.0419)
Landownership	0.0162 (0.0299)	0.2688 (0.2814)	0.0151 (0.0254)	-1.0081 (1.4200)
Interest rate	-0.3294 (0.0045)∗∗∗	-0.1613 (0.0610)∗∗∗	-0.2896 (0.0052)∗∗∗	-3.4774 (0.6303)∗∗∗
Area of land (Log)	0.0419 (0.0124)∗∗∗	1.4448 (0.1504)∗∗∗	-0.0080 (0.0102)	-0.1749 (0.6754)
Access to extension	0.0058 (0.0134)	-0.0788 (0.1458)	0.0056 (0.0114)	-0.2378 (0.8655)
Labour	-0.0065 (0.0119)	-0.1557 (0.1245)	0.0015 (0.0101)	-0.7137 (0.7762)
Dadu	-0.0752 (0.0226) ∗∗∗	0.2052 (0.214)	-0.0619 (0.0194)∗∗∗	-2.4016 (1.2273)∗
Shikarpur	-0.0667 (0.0229) ∗∗∗	0.1349 (0.2607)	-0.0592 (0.0196)∗∗∗	-3.2659 (3.2400)
Jacobabad	-0.1091 (0.0221)∗∗∗	-0.4262 (0.2105)∗∗	-0.0869 (0.0188)∗∗∗	-4.5257 (1.5495)∗∗∗
Nawabshah	-0.0283 (0.0228)	0.7523 (0.2353)∗∗∗	-0.0713 (0.0193)∗∗∗	-4.4293 (1.9358)∗∗
Sanghar	-0.0171 (0.0234)	1.0224 (0.2716)∗∗∗	-0.0633 (0.0198)∗∗∗	-2.6189 (1.2781)∗∗
Income	-0.1353 (0.0204)∗∗∗	-	0.0369 (0.0157)∗∗	2.0242 (1.0848)∗
Collateral	-	0.6983 (0.1747)∗∗∗	-0.1786 (0.0159)∗∗∗	-4.5171 (1.2262)∗∗∗
Constant	1.1262 (0.1101)∗∗∗	0.3469 (1.1305)	0.9765 (0.0934)∗∗∗	13.9770 (7.0538)∗∗
/Athrho∗		0.8872 (0.0966)∗∗∗		
/lnsigma	-1.8897 (0.0340)∗∗∗			
Rho		0.7100 (0.0477)		
Sigma		0.1511 (0.0051)∗∗∗		
Chi^2^ = 24.33	LR test of independence equations:Prob > Chi2 = 0.000			

Standard Errors are reported in parenthesis as. ∗∗∗, ∗∗and ∗denotes 1, 5 and 10 statistically significant levels.

∗lnsigma and Athrho are transformations of sigma and rho that are used in' the estimation process. Source: Field survey 2017.
